# LncRNA ENSMUST00000171502 Induced by HIF-1α Ameliorates Ischemic Acute Kidney Injury via Targeting the miR-130b-3p/Mybl-1 Axis

**DOI:** 10.3390/cells11233747

**Published:** 2022-11-23

**Authors:** Jinghong Xu, Bing Wang, Dongshan Zhang

**Affiliations:** 1 Department of Emergency, Second Xiangya Hospital, Central South University, Changsha 410011, China; 2 Emergency Medicine and Difficult Diseases Institute, Second Xiangya Hospital, Central South University, Changsha 410011, China; 3 Department of Spine Surgery, Second Xiangya Hospital, Central South University, Changsha 410011, China; 4 Department of Nephrology, Second Xiangya Hospital, Central South University, Changsha 410011, China

**Keywords:** AKI, lncRNA171502, miRNA-130b-3p, Mybl-1, apoptosis

## Abstract

**Background:** Numerous studies have suggested that long non-coding RNA (lncRNA) affects the progression of ischemic acute kidney injury (IAKI). However, little information is currently available concerning the mechanisms of lncRNA171502 involved in IAKI. **Methods:** We applied an RT-qPCR assay for the expression of lncRNA171502 and miRNA-130b-3p, immunoblotting for the detection of Mybl-1-myeloblastosis oncogene-like 1 (Mybl-1) and cleaved caspase-3 (CC3) expression, and flow cytometry (FCM) for the evaluation of apoptosis. **Result:** Initially, lncRNA171502 was induced by HIF-1α in the mouse proximal tubular (BUMPT) cell line and C57BL/6J mice during ischemic injury. Secondly, ischemic injury-induced BUMPT cell apoptosis was markedly relieved following the overexpression of lncRNA171502. However, this effect was enhanced by the knockdown of lncRNA171502. Mechanistically, lncRNA171502 could sponge miRNA-130b-3p and would subsequently upregulate the expression of Mybl-1 to drive the apoptotic process. Lastly, the overexpression of lncRNA171502 alleviated the development of IAKI by targeting miRNA-130b-3p/Mybl-1 pathways. **Conclusions:** In summary, the HIF-1α/lncRNA171502/miRNA-130b-3p/Mybl-1 axis prevented the progression of IAKI and might serve as a potential therapeutic target.

## 1. Introduction

Ischemic/reperfusion injury (IRI) induces acute kidney injury (AKI), ischemic stroke, acute coronary syndrome, and circulatory arrest, which lead to high morbidity and mortality [1]. Some patients with ischemic AKI (IAKI) eventually progress into chronic renal failure or even end-stage renal failure [2,3,4]. Hence, IAKI has received considerable attention. Over the past two decades, researchers have commonly recognized that the apoptosis, necrosis, and inflammation of renal cells caused by IRI could contribute to the progression of AKI [5,6,7,8,9]. However, the mechanisms of renal cell apoptosis remain largely unexplored.

Long non-coding RNA (lncRNA) is an RNA transcript greater than 200 nucleotides in length. lncRNA plays vital roles in many physiological processes and acts as a competing endogenous RNA (ceRNA) to modulate microRNA (miRNA) activities [10,11]. MiRNA, especially 22 nt endogenous RNA, targets mRNA to regulate gene expression via the suppression of translational activity [12]. In recent years, many studies have demonstrated that lncRNA is associated with the progression of AKI. For example, lncRNAs MEG3, XIST, and ENSMUST_147219 mediated IAKI by facilitating the apoptosis of renal tubular cells [13,14,15]. By contrast, H19, lncRNA136131, and lncRNA NONRATG019935.2 mitigated IAKI via the suppression of renal tubular epithelial cell apoptosis [16,17,18]. LncRNA171502 is located at chr14:41307367-41307568. However, its function and regulatory mechanisms in IAKI remain unclear and require further investigation.

Here, we report that high expression of lncRNA171502 was found in an HIF-1α-induced, mouse proximal tubule-derived cell line (BUMPT) and in C57BL/6J mice after ischemic injury. LncRNA171502 could alleviate the I/R-induced apoptosis of BUMPT cells via the regulation of the miR-130b-3p/Mybl-1 axis. Finally, ischemic AKI might be alleviated by the overexpression of lncRNA171502.

## 2. Materials and Methods

### 2.1. Regents and Antibodies

The following reagents were used in this study: antibodies against cleaved C3 (CC3, Cat#9661s) and HIF-1α (Cat#36169; Cell Signaling Technology, Danvers, MA, USA); caspase-3 (C3, Cat#ab184787; Abcam, Waltham, MA, USA); Mybl-1 (Abcam) and β-tubulin (Cat#T0023; Affinity Biosciences, Cincinnati, OH, USA); lncRNA171502 siRNA, miRNA-130b-3p inhibitor, miRNA-130b-3p mimic, Mybl-1 siRNA, and lncRNA171502 plasmid (Ruibo, Guangzhou, China); TRIzol reagent (Invitrogen, Waltham, MA, USA); lipofectamine 2000 (Life Technologies, Carlsbad, CA, USA); fluorescein isothiocyanate (FITC) Annexin V Apoptosis Detection Kit I (Cat#556547; BD Pharmingen, San Jose, CA, USA); luciferase assay kit (BioVision, Waltham, MA, USA); Ag SYBR Green Pro Taqhs premix (Accurate Biotechnology Co., Ltd., Changsha, China); antimycin A (mitochondrial complex III inhibitor, Cat#ab141904; Abcam, Cambridge, UK); and calcium ion carrier (Cat#A23187; Sigma, St. Louis, MO, USA).

### 2.2. Cell Culture and Treatments

BUMPT cells were cultured with DMEM (Sigma-Aldrich, St. Louis, MO, USA) containing 10% FBS and antibiotics at 37 °C with 5% CO_2_. When the cell density reached about 90%, the ATP depletion cell model was subjected to antimycin A (10 μM) and calcium ionophore (1.5 μM), as previously described [19]. The lipofectamine 2000 was applied for transfection of lncRNA171502 siRNA or plasmid, miRNA-130b-3p mimic, miRNA-130b-3p inhibitor, Mybl-1 siRNA, or the negative control.

### 2.3. Establishment of Ischemic AKI Model

The C57BL/6J mice (male, 8–10 weeks) obtained from the Shanghai Animal Center (Shanghai, China) were kept under a 12 h light/dark cycle and provided unlimited access to water and food. The lncRNA171502 plasmid (25 μg per injection) was given to each male C57BL/6J mouse via tail-vein injection 24 h before ischemia–reperfusion injury [20]. The renal blood supply was blocked for 30 min followed by 24 or 48 h of reperfusion [21].

### 2.4. RT-qPCR Analysis

Total RNA was extracted from BUMPT cells and mouse kidney cortex with Trizol, and 1 μg of it was reversed to single-stranded DNA by using Evo M-MLV. For the cytoplasmic and nuclear RNA separation, BUMPT cell fractionation and purification were conducted using the Cytoplasmic and Nuclear RNA Purification Kit (Norgen, #21000,37400) [22]. RT-qPCR was carried out using the LightCycler^®^ 480 II (Basel, Switzerland). Ag SYBR Green Pro Taqhs premix was utilized according to the kit’s instruction. The sequences of lncRNA171502 were obtained from the Ensembl database (Gen ID: ENSMUST00000171502). The sequences of each primer pair were as follows: lncRNA171502, 5′-AATGTGATGCTCCGTTGCCAGTC-3′ (F), and 5′-CCATCTTCCTCCCACTCCTCCAAG-3′ (R); miRNA-130b-3p, 5′-GCGCAGTGCAATGATGAAA-3′ (F), and 5′-AGTGCAGGGTCCGAGGTATT-3′ (R); Mybl-1, 5′-GTCGTAATGGTGGAGACAGTGAAGC-3′ (F), and 5′-TCTGAGGATGGTTGGAGGAGTGC-3′ (R); HIF-1α, 5′-ACCACAACTGCCACCACTGATG-3′ (F), and 5′-TGCCACT GTATGCTGATGCCTTAG-3′ (R); β-actin, 5′-GGCTGTATTCCCCTCCATCG-3′ (F), and 5′-CCAGTTGGTAACAATGCCATGT-3′ (R); U6, 5′-CTCGCTTCGGCAGCACA-3′ (F), and 5′-AACGCTTCACGAATTTGCGT-3′ (R). Chip-PCR analysis was conducted with specific primers F/R: (5′-GGACAAGGACTGAAGGAACAGAAGG-3′ (F) and 5′-CCCCTCATGTACTATGTGCTTGGTG-3′ (R)). All of the primer pairs were supplied by Sangon Biotech (Shanghai, China). The Ct values obtained from different samples were compared using the 2^−ΔΔCt^ method.

### 2.5. ChIP Analysis

The binding site of HIF-1α interacting with the promoter of lncRNA171502 was analyzed in accordance with the protocol of a commercial kit (Millipore, Burlington, MA, USA). Briefly, samples were subjected to ultrasonic treatment. Then, the supernatant was collected for immunoprecipitation with anti-HIF-1α antibody for PCR. A PCR analysis was conducted with the specific primer pair: 5′-GGACAAGGACTGAAGGAACAGAAGG-3′ (F) and 5′-CCCCTCATGTACTATGTG CTTGGTG-3′ (R).

### 2.6. Immunoblot Analysis

Western blotting was performed as described previously [23,24,25]. The whole cell and renal cortex lysates were separated through SDS/PAGE and then transferred onto nitrocellulose membranes. After incubation with C3, CC3, HIF-1α, and Mybl-1, the membranes were further incubated with the secondary antibodies. Finally, the protein blots were visualized with enhanced chemiluminescence reagent.

### 2.7. FISH Analysis

The fluorescent probes of lncRNA171502 and miRNA-130b-3p, U6, and 18S were synthesized by Ruibo company (Guangzhou, China). The nucleus and cytoplasm of BUMP cells were stained with U6 and 18S, respectively, and lncRNA171502 was labeled with Cy3. The sections of mouse kidney and BUMPT cells were hybridized with the associated probes for 24 h, followed by 4,6-diamino-2-phenylindole staining. Fluorescence imaging was carried out using a laser scanning confocal microscope.

### 2.8. FCM Analysis of Apoptosis

Apoptosis was examined by annexin V-FITC/PI staining. Briefly, BUMPT cells were collected and resuspended with binding buffer and then incubated in the dark for 15 min after annexin V-FITC staining and 5 min of PI staining. The binding buffer (200 uL) was added to detect cell apoptosis using the Annexin-V-FITC apoptosis detection kit (Cat#556547; BD Pharmingen). The apoptotic rates were measured as the total percentage of cells that underwent advanced apoptosis (Annexin V+/PI+) and early apoptosis (AnnexinV+/PI−) [19].

### 2.9. Luciferase Reporter Assays

As previously described, DLR assays were used in this study. DLRs of wild-type and mutated plasmids of WT-Luc-Mybl1, WT-Luc-lncRNA171502, MUT-Luc-Mybl-1, and MUT-Luc-lncRNA171502 were constructed and then co-transfected with miRNA-130b-3p mimic or scrambled into BUMPT cells for 48 h [26,27,28]. Subsequently, luciferase activities were assessed by SpectraMax M5 (Molecular Devices, San Jose, CA, USA) after normalization to pGMLR-TK.

### 2.10. Renal Function and Morphology

The concentrations of serum creatinine (Cr) and urea nitrogen (BUN) were examined by a renal function examination kit. The morphology of renal tissues was assessed by hematoxylin and eosin (H&E) staining [29,30]. TUNEL staining was applied to observe cell apoptosis [23]. Tissue damage was scored as follows: 4, >75% damage; 3, 50–75% damage; 2, 25–50% damage; 1, <25% damage; 0, no damage.

### 2.11. Statistical Analysis

The two group comparisons were conducted utilizing the two-tailed Student’s *t*-tests. The differences among multiple groups were compared using one-way ANOVA. The data with non-normal distributions were analyzed using the Kruskal–Wallis test. For the apoptosis ration using FCM, chi-square or Fisher’s exact test was utilized. Statistical tests were conducted with the GraphPad software 8.0 (Windows GraphPad Software, San Diego, CA, USA). Quantitative data are expressed as mean ± SD. The statistical significance was set at *p* < 0.05.

## 3. Results

### 3.1. The Expression of lncRNA171502 Is Upregulated by IRI in BUMPT Cells and Mouse Kidney

**Figure 1:**
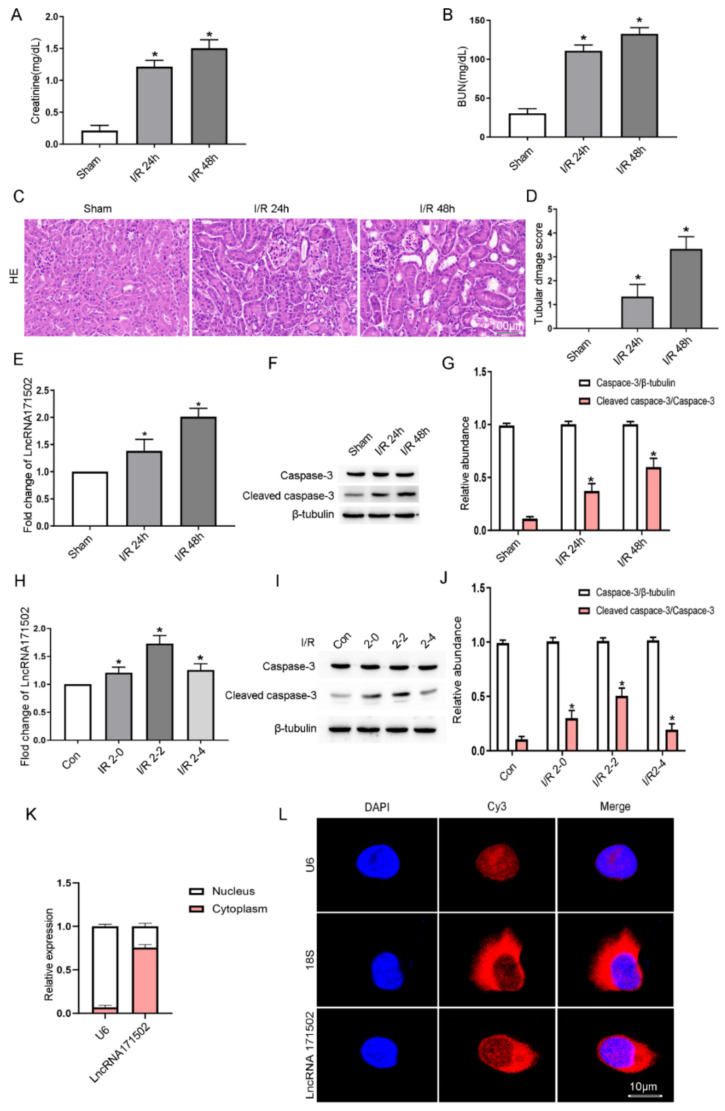
**The effect of IRI on lncRNA171502 expression in C57/BL6 mice and BUMPT cells.** C57/BL6 mice were exposed to ischemia (I, 30 min)/reperfusion (R, 24 or 48 h), respectively. The levels of Cr (**A**) and BUN (**B**). (**C**) Then, H&E staining was carried out. Score bar: 100 μm. (**D**) The tubular damage score was evaluated. (**E**) Additionally, RT-qPCR was applied for the observation of lncRNA171502 expression. (**F**) Immunoblot assessment of the levels of apoptosis-related proteins. (**G**) Densitometric analysis of C3, CC3, and β-tubulin were carried out. In addition, BUMPT cells were exposed to 1.5 μM calcium and 10 μM antimycin A (I, 2 h)/(R, 0, 2, and 4 h). (**H**) RT-qPCR was used for the measurement of lncRNA171502 expression in BUMPT cells. (**I**) Immunoblot assessment of the levels of C3 and CC3. (**J**) Densitometric analysis of C3, CC3, and β-tubulin. (**K**) RT-qPCR was used for the intracellular localization of lncRNA171502; U6 was used as a control for nuclear marker. (**L**) RNA-FISH examination of the intracellular localization of lncRNA171502 in BUMPT cells. U6 was employed as a control for nuclear marker, whereas 18S was used as control for cytoplasmic marker, Score bar is 10 μm. Mean ± SD (*n* = 6). * *p* < 0.05, IRI group vs. sham or control group.

In an attempt to determine whether IRI can increase the expression of lncRNA171502, the C57/BL6 mice were exposed to I (30 min)/R (24 or 48 h). The renal function displayed that the serum levels of Cr and BUN were elevated at 24 h after reperfusion and attained a peak at 48 h after reperfusion (Figure 1A,B). The results of H&E staining and tubular injury score at 24 and 48 h after reperfusion revealed a decline in renal function (Figure 1C,D). Furthermore, RT-qPCR analysis indicated that the mRNA expression of lncRNA171502 was gradually upregulated at 24 h after reperfusion and peaked at 48 h (Figure 1E). The immunoblot results of CC3 were in agreement with the expression of lncRNA171502 at the indicated time points (Figure 1F,G). In addition, the findings of RT-qPCR and immunoblotting revealed that the protein and mRNA expression levels of lncRNA171502 and CC3 were also upregulated after antimycin treatment; they peaked at 2 h and then decreased (Figure 1H–J). RT-qPCR and FISH experiments demonstrated that lncRNA171502 was localized in the cytoplasm of BUMPT cells (Figure 1K,L). The data suggest that lncRNA171502 is responsible for the progression of ischemic injury.

### 3.2. The Expression of lncRNA171502 Is Mediated by HIF-1α

**Figure 2:**
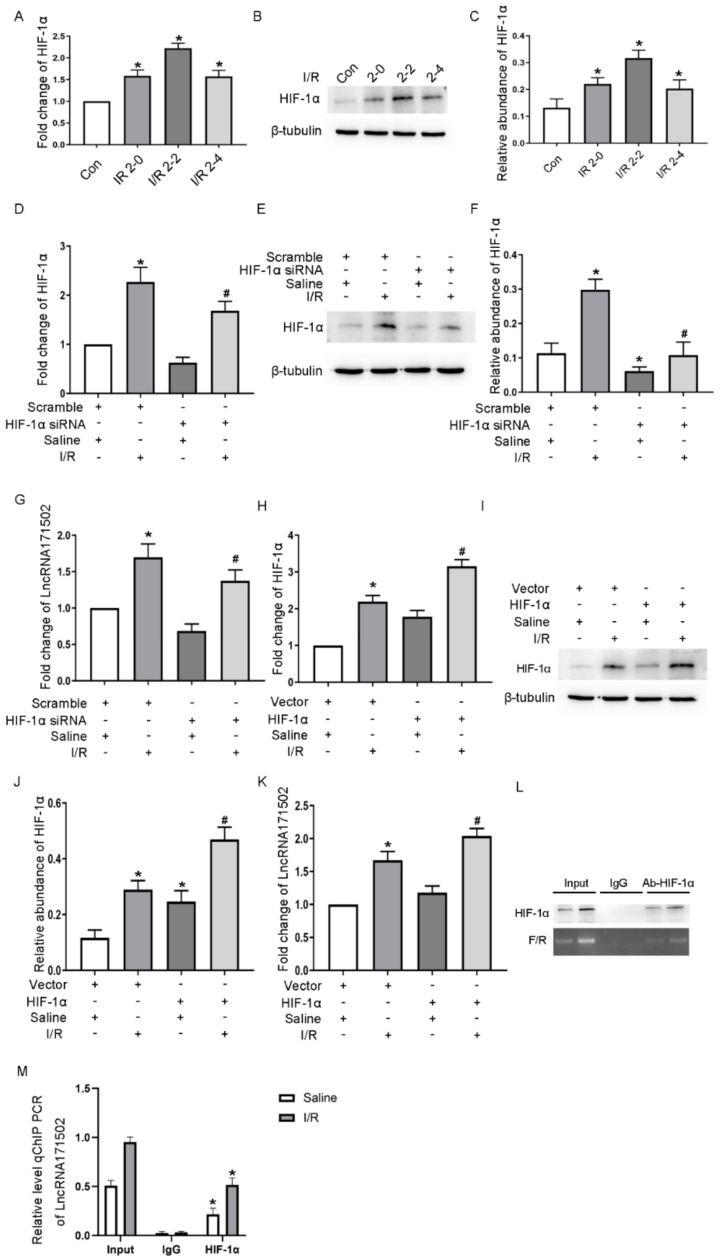
IRI-induced expression of HIF-1α regulates the expression of lncRNA171502 in BUMPT cells. The cells were given 1.5 μM calcium and 10 μM antimycin A (I, 2 h)/(R, 0, 2, and 4 h), transfected with HIF-1α siRNA/plasmid or scramble, and subsequently treated with/without I (2 h)/R (2 h) injury. (**A**) RT-qPCR was applied for the determination of the HIF-1α level. (**B**) Immunoblot assessment of HIF-1α expression. (**C**) Gray determination of HIF-1α and β-tubulin. (**D**) RT-qPCR for the detection of the HIF-1α level. (**E**) Immunoblot evaluation of HIF-1α expression. (**F**) Densitometric analysis of HIF-1α and β-tubulin. (**G**) RT-qPCR for the measurement of the lncRNA171502 level. (**H**) RT-qPCR for the assessment of HIF-1α expression. (**I**) Immunoblot assessment of the HIF-1α level. (**J**) Densitometric analysis of HIF-1α and β-tubulin. (**K**) RT-qPCR for the detection of the lncRNA171502 level. (**L**) Chip detection revealed a binding site (121 bp fragment) for HIF-1α in the promoter region of lncRNA171502. The two lanes mean control and IRI. Input means to detect the HIF-1α protein in the promoter region of lncRNA502 without ChIP; IgG refers to the negative control in the ChIP process to ensure the removal of false positives; Ab-HIF-1α means the detection of HIF-1α in the promoter region of lncRNA502 after ChIP. (**M**) ChIP analysis of HIF-1α binding to lncRNA171502 promoter. The immunoprecipitated samples were subjected to RT-qPCR analysis of lncRNA171502 promoter sequences. Mean ± SD (*n* = 6). * *p* < 0.05, IRI group vs. sham or control group. ^#^ *p* < 0.05, HIF-1α siRNA group with IRI or HIF-1α plasmid group with IRI vs. scramble group with IRI.

We investigated whether HIF-1α promoted the expression of lncRNA171502 during ischemic injury. The protein and mRNA expression levels of HIF-1α were raised at 0 h, peaked at 2 h, and diminished after antimycin treatment (Figure 2A–C). Meanwhile, RT-qPCR or Western blot analysis revealed that the mRNA and its protein levels, along with lncRNA171502 levels under basal and I/R conditions, were suppressed by the expression of HIF-1α (Figure 2D–G). Additionally, this effect was reinforced after the overexpression of HIF-1α (Figure 2J,K). Using the Jaspar core database (http://jaspar.Genereg.net/, accessed on 7 September 2022), the predication results showed that the lncRNA171502 promotor sequence contained one binding site of HIF-1α. Chip detection indicated that HIF-1α binds to the site (121 bp fragment) of the promoter regions of lncRNA171502 (Figure 2L,M). Hence, the data indicated that HIF-1α was linked to lncRNA171502 during I/R.

### 3.3. LncRNA171502 siRNA Promotes IRI-Induced BUMPT Cell Apoptosis

**Figure 3:**
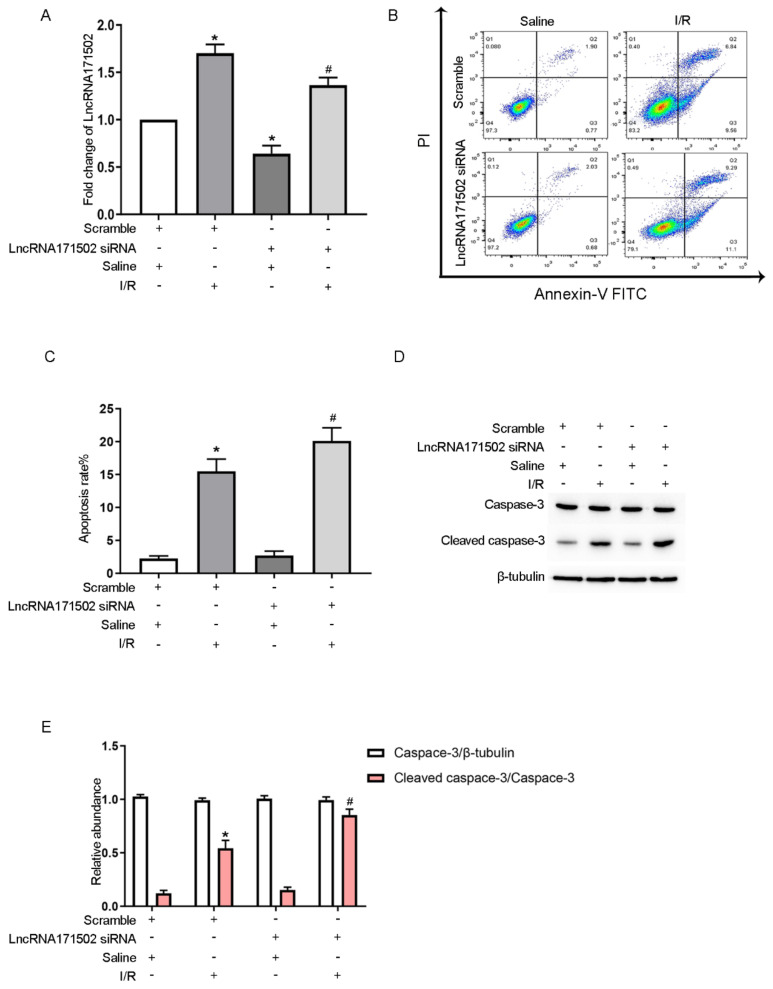
**LncRNA171502 siRNA facilitated IRI-induced BUMPT cell apoptosis**. After transfection with lncRNA171502 siRNA (100 nM) or scramble, the cells were exposed to I (2 h)/R (2 h) injury. (**A**) RT-qPCR was utilized for the examination of the lncRNA 171502 level. (**B**) FCM for the observation of BUMPT cell apoptosis. (**C**) Representative apoptotic rate (%). (**D**) Immunoblotting was applied to measure the expression levels of apoptosis-related proteins. (**E**) Densitometric analysis of C3, CC3, and β-tubulin. Mean ± SD (*n* = 6). * *p* < 0.05, scramble or lncRNA 171502 siRNA group with IRI vs. scramble group; ^#^ *p* < 0.05, lncRNA 171502 siRNA group with IRI vs. scramble group with IRI.

The effect of lncRNA171502 on BUMPT cell apoptosis induced by IRI was analyzed. RT-qPCR assays indicated that lncRNA171502 siRNA inhibited its expression level under the basal and IRI conditions (Figure 3A). FCM analysis revealed that IRI-induced BUMPT cell apoptosis was promoted after exposure to lncRNA171502 siRNA (Figure 3B,C). In addition, the immunoblotting data of CC3 supported the FCM results (Figure 3D,E). Collectively, these results demonstrate that lncRNA171502 exerts an anti-apoptosis function during IRI.

### 3.4. Upregulation of lncRNA171502 Attenuates IRI-Induced BUMPT Cell Apoptosis

**Figure 4:**
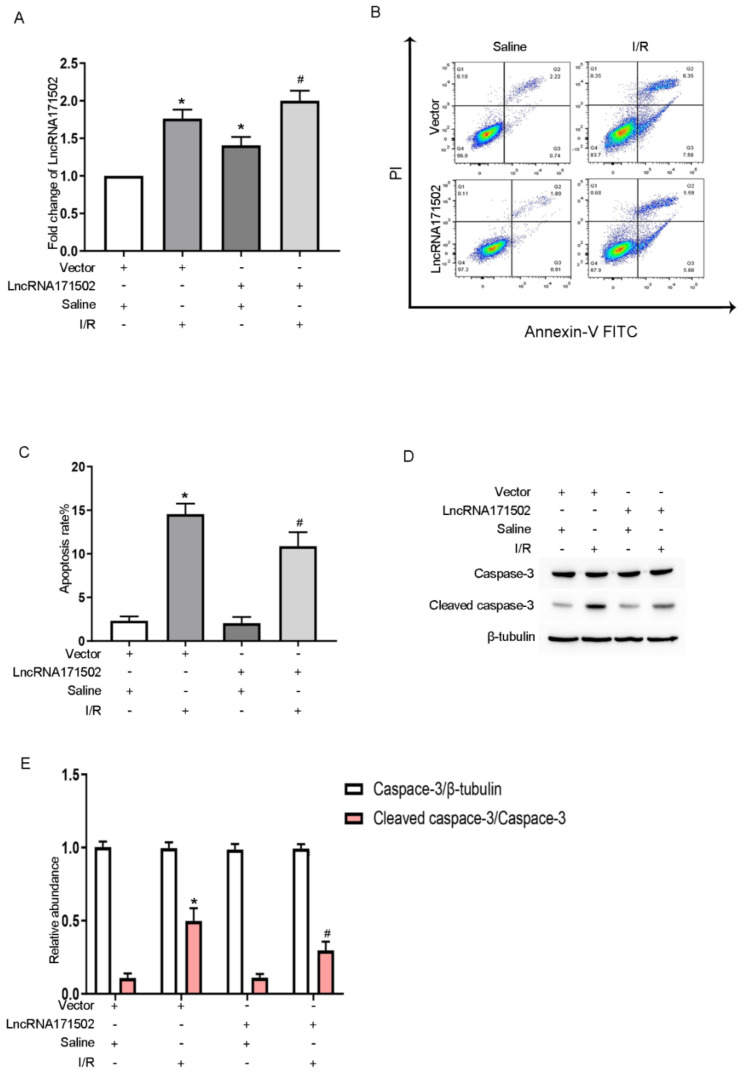
**Overexpression of lncRNA171502 attenuates IRI-induced BUMPT cell apoptosis.** BUMPT cells were exposed to cell transfection with lncRNA171502 plasmid or control, followed by exposure to I (2 h)/R (2 h) injury or not. (**A**) RT-qPCR was used to detect the lncRNA 171502 level. (**B**) FCM was applied to analyze BUMPT cell apoptosis. (**C**) Representative apoptotic rate (%) was assessed. (**D**) Immunoblotting was utilized to examine the levels of C3 and CC3. (**E**) Densitometric analysis of C3, CC3, and β-tubulin. Mean ± SD (*n* = 6). * *p* < 0.05, scramble or lncRNA 171502 plasmid group with IRI vs. scramble group; ^#^ *p* < 0.05, lncRNA171502 plasmid group with IRI vs. scramble group with IRI.

We further determined whether lncRNA171502 elicits IRI-induced BUMPT cell apoptosis. The RT-qPCR assays indicated that the levels of lncRNA171502 were upregulated under the basal and IRI conditions after its overexpression (Figure 4A). The FCM analysis revealed that the overexpression of lncRNA171502 attenuated IRI-induced BUMPT cell apoptosis (Figure 4B,C). The immunoblotting results of CC3 were also consistent with the FCM findings (Figure 4D,E). Our data further verified the anti-apoptotic effect of lncRNA171502 during ischemic injury.

### 3.5. LncRNA171502 Sponges miRNA-130b-3p

**Figure 5:**
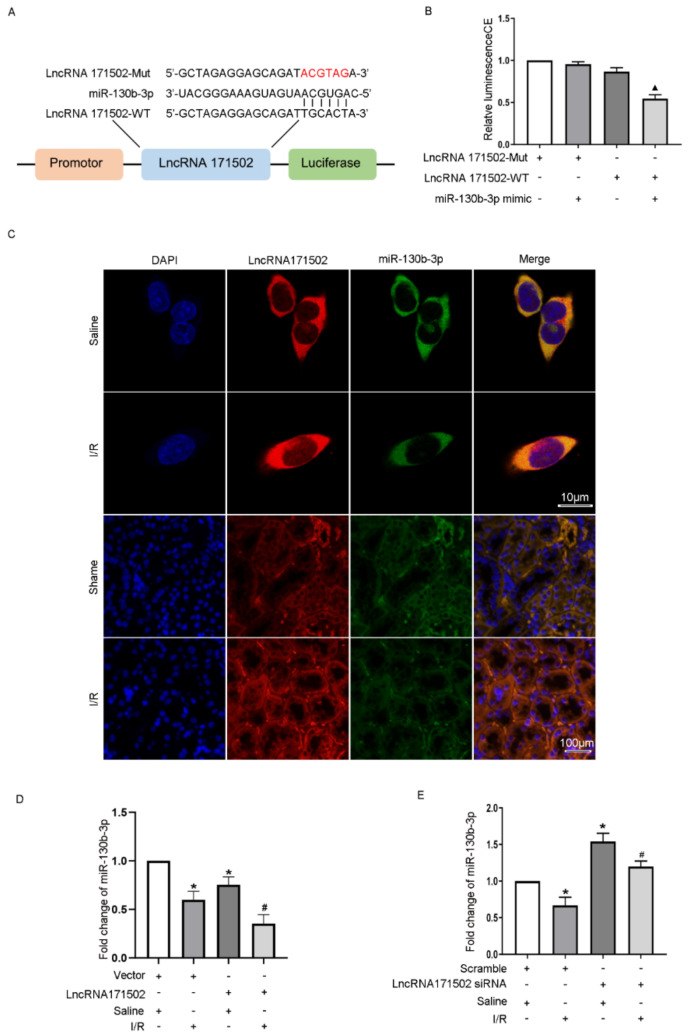
**LncRNA171502 suppresses the activity and expression of miRNA-130b-3p.** (**A**) The complementary sequences of lncRNA171502 and miRNA-130b-3p. (**B**) Following co-transfection of miRNA-130b-3p and lncRNA171502-WT or lncRNA171502-MUT, the luciferase activities were observed. (**C**) RNA-FISH analysis of the intracellular co-localization of lncRNA171502 and miRNA-130b-3p in BUMPT cells (Score bar: 10 μm) and renal tissues under the basal or IRI conditions (Score bar: 100 μm). (**D**,**E**) RT-qPCR determination of miRNA-130b-3p expression. Mean ± SD (*n* = 6). * *p* < 0.05, scramble group with IRI vs. scramble or vector group with saline; ^#^ *p* < 0.05, lncRNA 171502 siRNA or plasmid group with IRI vs. scramble group with IRI; ▲ *p* < 0.05, lncRNA171502 WT/miRNA-130b-3p vs. other groups.

Subsequently, the anti-apoptosis mechanism of lncRNA171502 was evaluated. The prediction findings obtained from the RegRNA 2.0 software demonstrated that lncRNA171502 consisted of the complementary sequences of miRNA-130b-3p (Figure 5A), suggesting that miRNA-130b-3p may be a potential target of lncRNA171502. The DLR analysis revealed that the luciferase activity of lncRNA171502-WT but not lncRNA171502-MUT was attenuated in the miRNA-130b-3p mimic group (Figure 5B). RNA-FISH experiments indicated that lncRNA171502 and miRNA-130b-3p co-localized in the cytoplasm of BUMPT cells and kidney tissues under the basal and IRI conditions (Figure 5C). Moreover, the overexpression of lncRNA171502 not only suppressed the expression of miRNA-130b-3p but also reinforced the inhibition of ischemic injury on the miRNA-130b-3p level. However, the effect of lncRNA171502 knockdown is the exact opposite of lncRNA171502 overexpression (Figure 5D,E). We also explored whether lncRNA171502 mutation can protect BUMPT cells from apoptosis induced by IRI (Supplementary Figure S1D–G). Taken together, these findings indicate that miRNA-130b-3p is a target of lncRNA171502.

### 3.6. IRI-Induced BUMPT Cell Apoptosis Is Accelerated by miRNA-130b-3p Mimics

**Figure 6:**
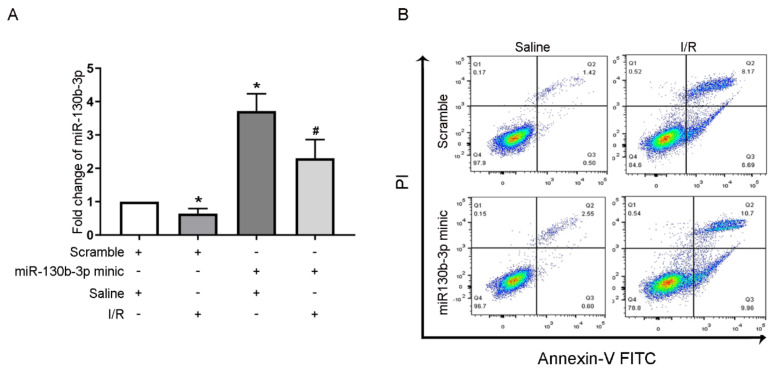

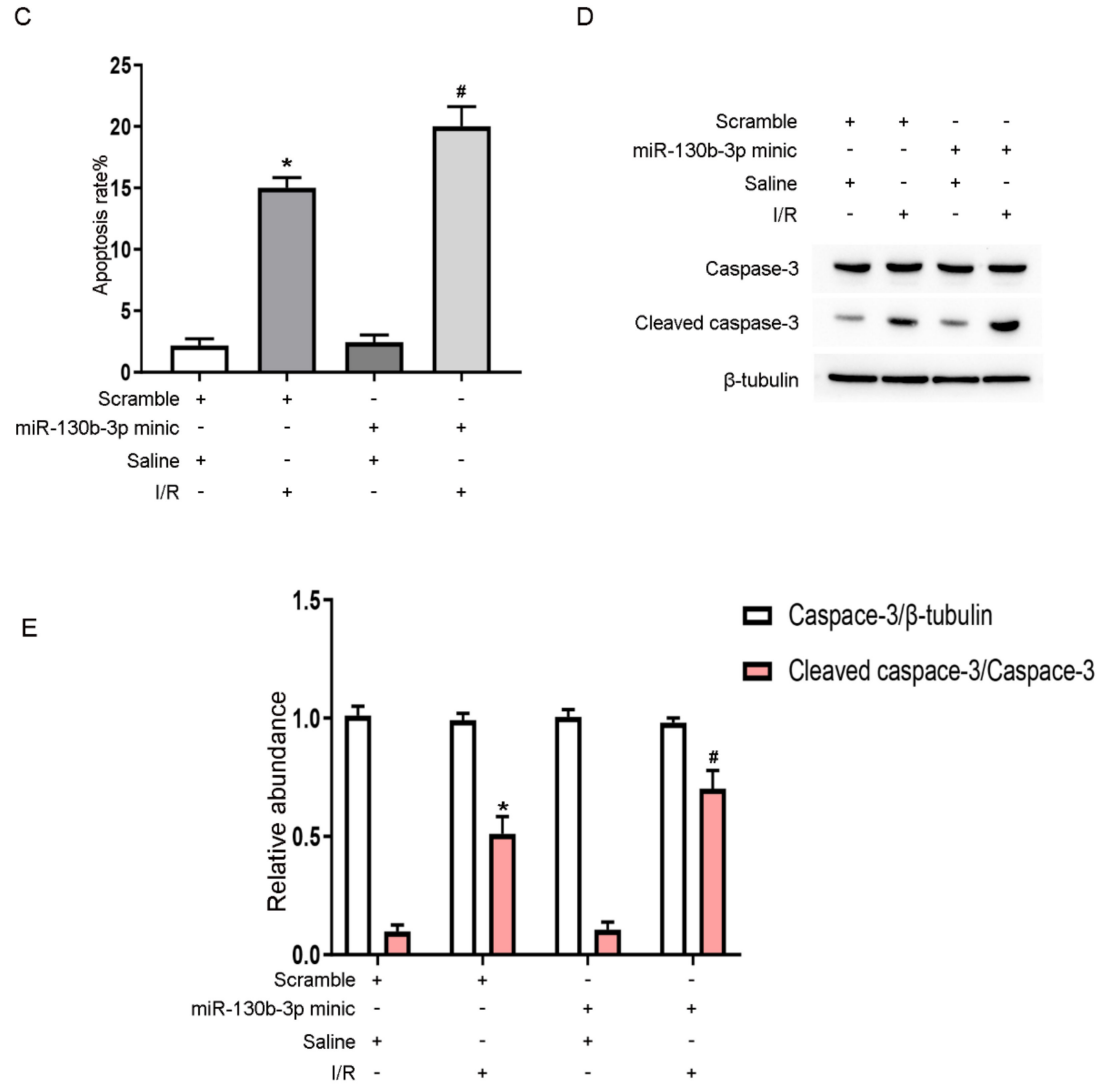
**Overexpression of miRNA-130b-3p elevates the levels of apoptosis-related proteins in BUMPT cells after IRI.** The cells were transfected with 100 nM miRNA-130b-3p mimics or scramble before I (2 h)/R (2 h) injury. (**A**) The expression of miRNA-130b-3p was evaluated by RT-PCR, (**B**) BUMPT cell apoptosis was determined by FCM, (**C**) and representative apoptotic rate (%) was calculated. (**D**) The levels of apoptosis-related proteins were detected by immunoblotting. (**E**) Densitometric analysis of C3, CC3, and β-tubulin. Mean ± SD (*n* = 6). * *p* < 0.05, scramble group with IRI or mimic group with saline vs. scramble group; ^#^ *p* < 0.05, miRNA-130b-3p mimic group with IRI vs. scramble group with IRI.

A recent study has demonstrated that miRNA-130b-3p can mediate IRI-induced cardiomyocyte apoptosis [31]. The RT-qPCR assays revealed that the miRNA-130b-3p mimics potentiated miRNA-130b-3p’s expression under the basal and IRI conditions (Figure 6A). The FCM and immunoblot analyses indicated that transfection with miRNA-130b-3p mimics promoted IRI-induced apoptosis and enhanced the expression of CC3 in BUMPT cells (Figure 6B–E). Taken together, these results showed that miRNA-130b-3p could also promote the apoptosis of renal cells during ischemic injury.

### 3.7. Mybl-1 Is a Target Gene of miRNA-130b-3p

**Figure 7:**
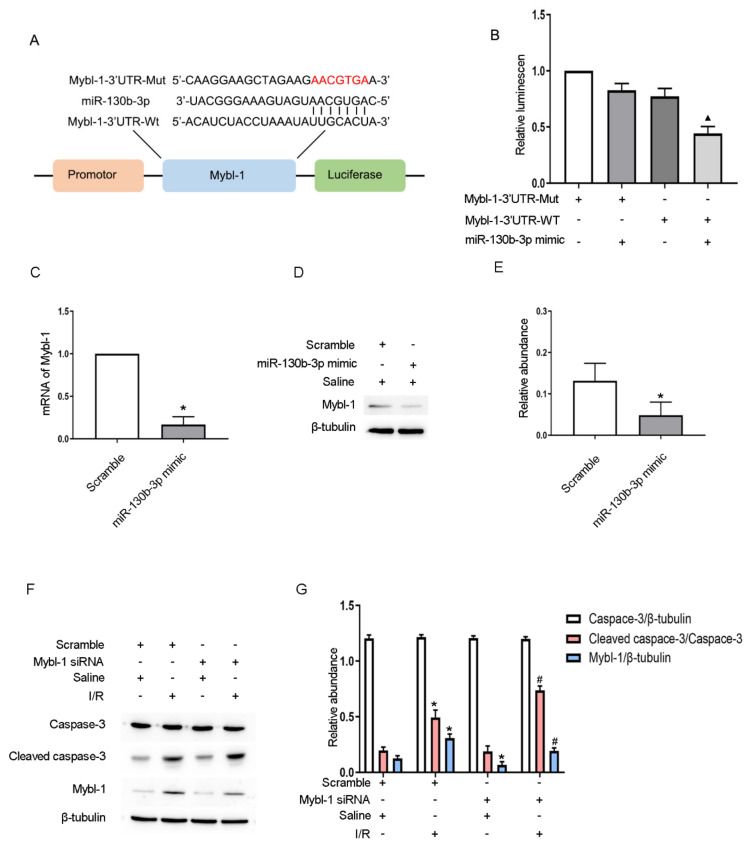
**Mybl-1 is a direct target of miRNA-130b-3p.** The cells were exposed to 100 nM miRNA-130b-3p mimic or 100 nM Mybl-1 siRNA or scramble before I (2 h)/R (2 h) injury. (**A**) The complementary sequences of miRNA-130b-3p and Mybl-1. (**B**) miRNA-130b-3p was co-transfected with the 3′UTR DLR vector of Mybl-1-WT or MUT, and then the luciferase activities were detected. (**C**) RT-qPCR determination of the mRNA level of Mybl-1. (**D**) Immunoblot assessment of the levels of Mybl-1 and β-tubulin. (**E**) Densitometric analysis of Mybl-1 and β-tubulin. (**F**) Immunoblot detection of C3, CC3, and Mybl-1 expression. (**G**) Densitometric analysis of C3, CC3, Mybl-1, and β-tubulin. Mean ± SD (*n* = 6). * *p* < 0.05, miRNA-130b-3p mimic group vs. scramble group or scramble group with IRI vs. scramble group with saline; ^#^ *p* < 0.05, Mybl-1 siRNA group with IRI vs. scramble group with IRI; ▲ *p* < 0.05, Mybl-1 WT/miRNA-130b-3p vs. other groups.

Mybl-1 is known as a member of the transcription factor MYB. It was found that miRNA-130b-3p contained the complementary sequences of Mybl-1 (Figure 7A,B). In addition, the protein and mRNA levels of Mybl-1 were remarkably inhibited by miRNA-130b-3p mimics, and Mybl-1 was closely related to ischemia–reperfusion (Figure 7C–E, Supplementary Figure S1A–C). The results of immunoblotting demonstrated that Mybl-1 siRNA further reinforced the IRI-induced expression of CC3 (Figure 7F,G). Our findings confirm that miRNA-130b-3p can promote cell apoptosis via targeting Mybl-1.

### 3.8. Deficiency of miRNA-130b-3p Reverses the Proapoptotic Effect of lncRNA171502 siRNA on BUMPT Cells during Ischemic Injury

**Figure 8:**
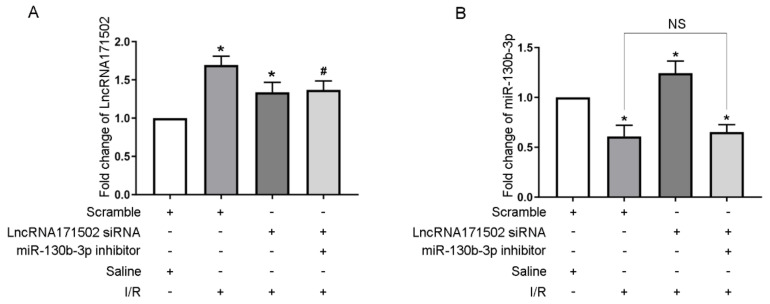

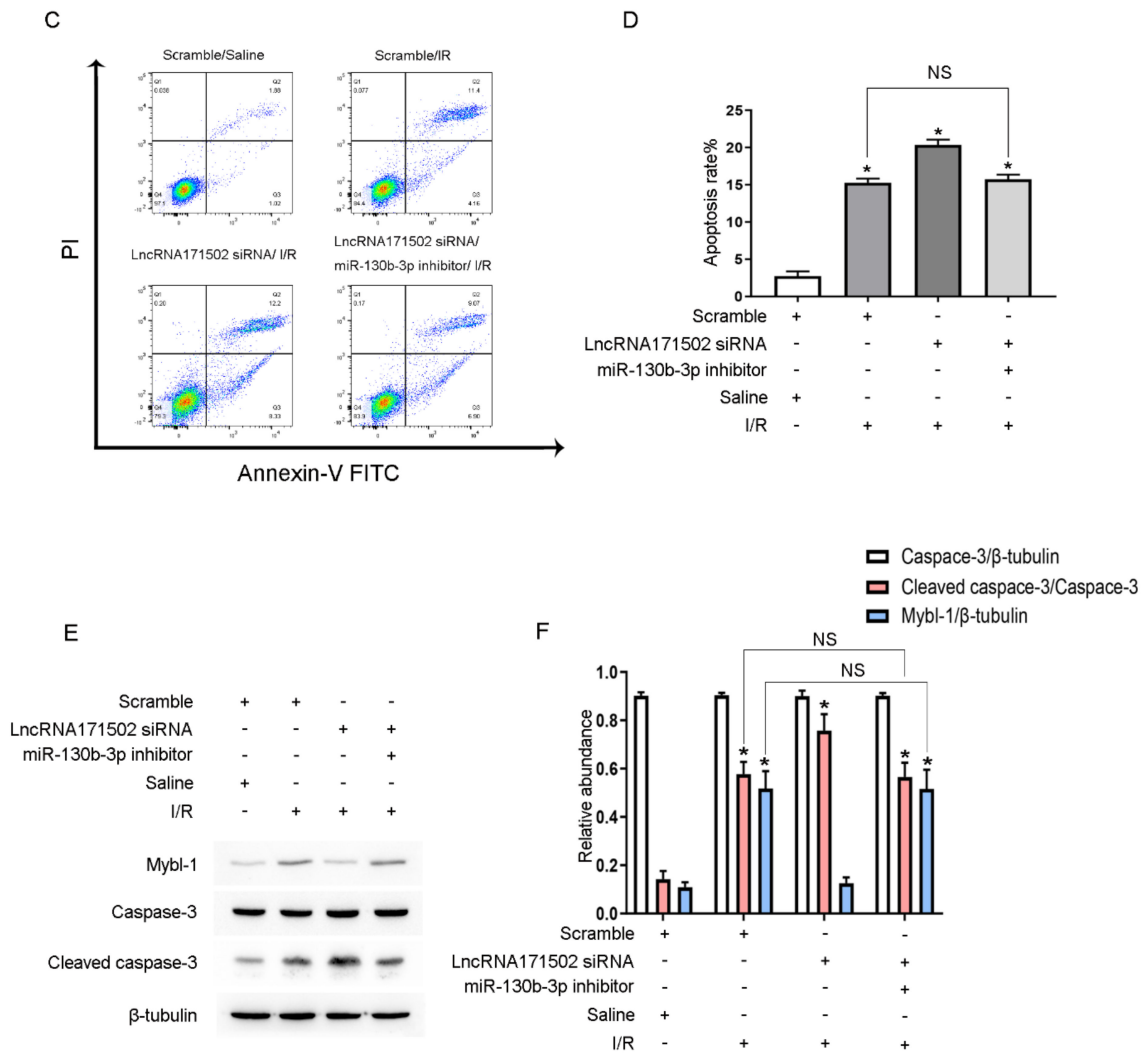
**miRNA-130b-3p inhibitor reverses the proapoptotic effect of lncRNA171502 siRNA during ischemic injury**. The cells were co-transfected with siRNA lncRNA171502 (100 nM) with/without anti-miRNA-130b-3p and then exposed to I (2 h)/R (2 h) injury. (**A**,**B**) The levels of lncRNA171502 and miRNA-130b-3p were measured by RT-qPCR. (**C**) BUMPT cell apoptosis was examined by FCM. (**D**) Representative apoptotic rate (%) was evaluated. (**E**) The levels of C3, CC3, and Mybl-1 were measured by immunoblotting. (**F**) Densitometric analysis of C3, CC3, Mybl-1, and β-tubulin. Mean ± SD (*n* = 6). * *p* < 0.05, scramble group with IRI vs. scramble group with saline; lncRNA171502 siRNA group with IRI vs. scramble group with IRI; ^#^ *p* < 0.05, lncRNA136131 siRNA + anti-miRNA-130b-3p group with IRI vs. scramble group with IRI. NS: no significance.

Furthermore, we determined whether miRNA-130b-3p is a key mediator of lncRNA171502. The RT-qPCR analysis confirmed that lncRNA171502 siRNA prominently diminished its level under the basal and IRI conditions, but transfection with the miRNA-130b-3p inhibitor did not affect its level (Figure 8A). The low expression of miRNA-130b-3p induced by IRI was reversed by lncRNA171502 siRNA, but this effect was subsequently repressed by exposure to the miRNA-130b-3p inhibitor (Figure 8B). The FCM and immunoblot analyses revealed that lncRNA171502 siRNA enhanced IRI-induced apoptosis and elevated the levels of Mybl-1 and CC3 in BUMPT cells. However, this effect was reversed after treatment with the miRNA-130b-3p inhibitor (Figure 8C–F). Our data confirm that miRNA-130b-3p is a key target of lncRNA171502.

### 3.9. IAKI Can Be Mitigated by lncRNA171502 Upregulation

**Figure 9:**
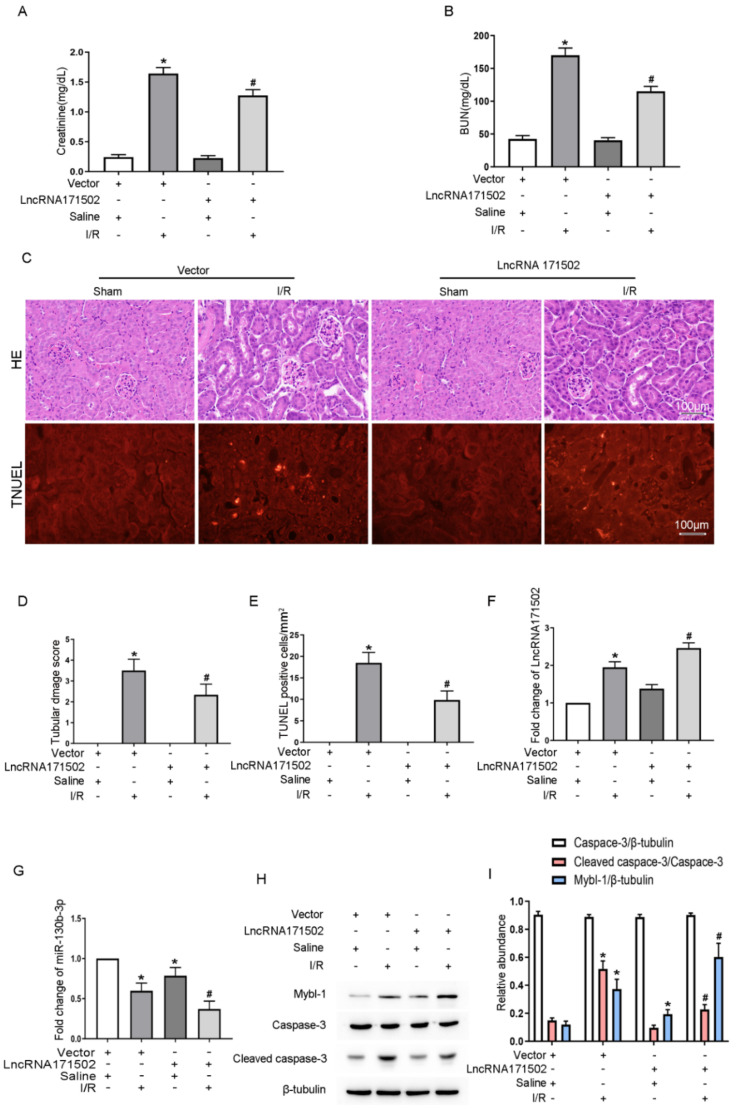
**Overexpression of lncRNA171502 alleviates IAKI.** The C57BL/6J mice were injected with lncRNA171502 plasmid via tail vein for 12 h and then exposed to I (30 min)/R (48 h). Blood serum was applied for examination of Cr (**A**) and BUN (**B**) concentrations. (**C**) Representative of H&E and TUNEL staining. Score bar: 100 μm. (**D**) Tubular damage scores of kidney cortex. (**E**) TUNEL-positive cells/mm^2^. (**F**,**G**) RT-qPCR measurement of the levels of lncRNA171502 and miRNA-130b-3p. (**H**) Immunoblot detection of C3, CC3, and Mybl-1. (**I**) Densitometric analysis of C3, CC3, Mybl-1, and β-tubulin. Mean ± SD (*n* = 6). * *p* < 0.05, vector group with IRI vs. sham group; ^#^ *p* < 0.05, lncRNA171502 plasmid group with IRI vs. vector group with IRI.

To corroborate the function of lncRNA171502 in IAKI, an lncRNA171502 plasmid or vector was injected via the tail vein of mice for 12 h, which then received I (30 min)/R (48 h). The lncRNA171502 plasmid significantly prevented the IRI-induced upregulation of serum Cr and BUN (Figure 9A,B). Furthermore, H&E and TUNEL staining also implicated that IRI-induced renal tubular damage and apoptosis were reduced by the lncRNA171502 plasmid, respectively (Figure 9C–E). The RT-qPCR assays indicated that lncRNA171502 plasmid elevated the lncRNA171502 level while reducing the expression of miRNA-130b-3p under the sham and IRI treatments (Figure 9F,G). Furthermore, the lncRNA 171502 plasmid decreased the IRI-induced expression levels of CC3 while elevating Mybl-1 expression (Figure 9H,I). Collectively, the data indicate that the lncRNA 171502/miRNA-130b-3p/Mybl-1 axis can attenuate the progression and development of IAKI.

## 4. Discussion

The function of lncRNAs in IAKI is still largely unclarified. In our study, we expounded that HIF-1α directly induced lncRNA171502 expression and then mitigated IRI-induced BUMPT cell apoptosis. Mechanistically, lncRNA171502 could act as a ceRNA to increase the expression of MYBL-1 by sponging miRNA-130b-3p. Therefore, the HIF-1α/lncRNA171502/miRNA-130b-3p/Mybl-1 axis is involved in the progression of IAKI, which explains the underlying molecular mechanism of this disease.

Accumulating studies have indicated that HIF-1α elicits a crucial effect on the regulation of miRNAs in various AKI models [32,33]. More recent studies reported that HIF-1α induced the expression of lncRNA NEAT1 in contrast-induced AKI [34]. Here, we also found that HIF-1α directly mediated the expression of lncRNA171502 caused by IRI (Figure 2). In terms of its mechanism, HIF-1α bound to the promoter region of lncRNA171502, thus promoting the expression of lncRNA171502. Another significant finding of this study was that lncRNAs could modulate IRI-induced renal tubular cell apoptosis. For example, the lncRNAs MEG3, XIST, and ENSMUST_147219 are apoptosis inducers [13,14,15]. However, H19, lncRNA136131, and lncRNA NONRATG019935.2 are apoptosis inhibitors [16,17,18]. The above studies suggest that the role of lncRNAs in apoptosis needs to be clarified during ischemic injury. In our study, we found that the overexpression or deficiency of lncRNA171502 could influence renal cell apoptosis caused by IRI exposure in vitro or in vivo (Figure 3, Figure 4 and Figure 9), implying that lncRNA171502 is an apoptosis inhibitor during ischemic injury.

**Figure 10:**
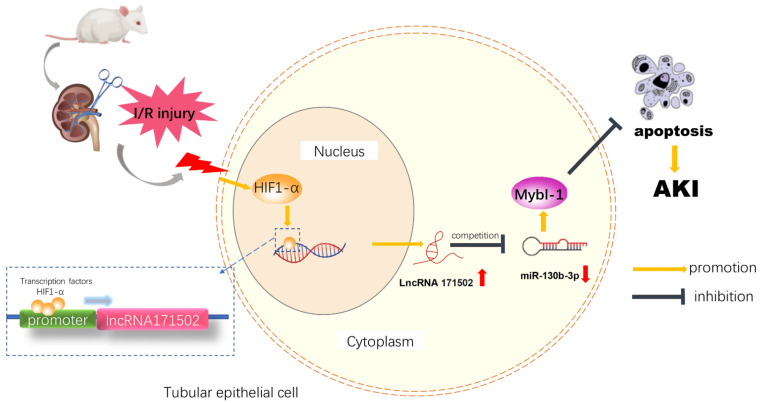
Graphical abstract showing how lncRNA ENSMUST00000171502 induced by HIF-1α ameliorates ischemic acute kidney injury via targeting miRNA-130b-3p/Mybl-1 pathways. In ischemia–reperfusion AKI, HIF-1α was induced by ischemia–reperfusion, bound to the promoter region of lncRNA171502, and promoted the expression of lncRNA171502. LncRNA171502 competitively sponged to miRNA-130b-3p, increased the expression of Mybl-1, and exhibited an anti-apoptotic role in both tubular epithelial cells and mouse models.

As we know, most LNRNAs act as ceRNAs to regulate the target gene expression [35]. Fox example, lncRNA MEG3 sponges miRNA-145-5p to increase the expression of RTKN [13]. LncRNA ENSMUST_147219 could sponge miRNA-221-5p to increase the expression of IRF6 [15]. In this study, we demonstrated that lncRNA171502 directly bound to miRNA-130b-3p, as evidenced by the prediction of RegRNA 2.0 software, DLR gene experiments, and their co-localization (Figure 5). Functionally, recent studies showed that miRNA-130b-3p mediated the apoptosis of cardiomyocytes during ischemic injury [31]. Consistently, we also found that miRNA-130b-3p could promote IRI-induced BUMPT cell apoptosis (Figure 6). Some studies have demonstrated that uncoupling protein 3 (Ucp3) and Forkhead box O3 (FOXO3) were direct target genes of miRNA-130b-3p [31,36]. Here, we reported that Mybl-1 was a new direct target of miRNA-130b-3p after DLR gene analysis and associated regulation experiments (Figure 7). Furthermore, some studies suggested that Mybl-1 was an apoptosis suppressor in tumor cells [37,38]. Consistently, we confirmed that the inhibition of Mybl-1 could enhance IRI-induced BUMPT cell apoptosis (Figure 7). Interestingly, rescue experiments verified that miRNA-103b-3p was a key downstream mediator of lncRNA171502 and reversed the proapoptotic effect of lncRNA171502 siRNA on BUMPT cells during ischemic injury (Figure 8). Finally, the lncRNA171502/miRNA-130b-3p/Mybl-1 axis was found to ameliorate the development of IAKI in vivo. Therefore, we believe that lncRNA171502 may serve as a novel therapeutic target for treating IAKI (Figure 9 and Figure 10).

## 5. Conclusions

This study, for the first time, indicated that lncRNA171502 was an apoptosis suppressor induced by HIF-1α during ischemic injury. Mechanistically, lncRNA171502 sponged miRNA-130b-3p to upregulate the expression of Mybl-1. The overexpression of lncRNA171502 could attenuate IAKI by regulating the miRNA-130b-3p/Mybl-1 axis. Taken altogether, the lncRNA171502/miRNA-130b-3p/Mybl-1 axis prevented the progression of IAKI and might be a new therapeutic target for treating this disease.

## Data Availability

All the data generated or analyzed during this study are included in this published article.

## Supplementary Materials

The following supporting information can be downloaded at: https://www.mdpi.com/article/10.3390/cells11233747/s1, Figure S1: The effect of I/R on the expression of Mybl-1 and LncRNA171502 mutation has no effect on ischemia induced BT cells apoptosis.

## Abbreviations

BUMPT—Boston university mouse proximal tubule; AKI—acute kidney injury; lncRNA—long non-coding RNA; I/R—ischemia/reperfusion; FCM—flow cytometry; BUN—blood urea nitrogen; FISH—fluorescence in situ hybridization; TUNEL—TdT-mediated dUTP nick end labeling; Mybl-1—myeloblastosis oncogene-like 1.
