# Supplementary material for: LncRNA ENSMUST00000171502 Induced by HIF-1α Ameliorates Ischemic Acute Kidney Injury via Targeting the miR-130b-3p/Mybl-1 Axis
Source: Cells. 2022 Nov 23;11(23):3747. doi: 10.3390/cells11233747 (PMC9735850; doi:10.3390/cells11233747)
Supplement: Supplementary file 1 [file cells-11-03747-s001.zip › cells-1931264-supplementary.pdf]

LncRNA ENSMUST00000171502 induced by HIF-1α ameliorates ischemic acute kidney injury via targeting the miR-130b-3p/Mybl-1 axis

Jinghong Xu [1,2,3], Bing Wang [3,*] and Dongshan Zhang [1,2,4,*]

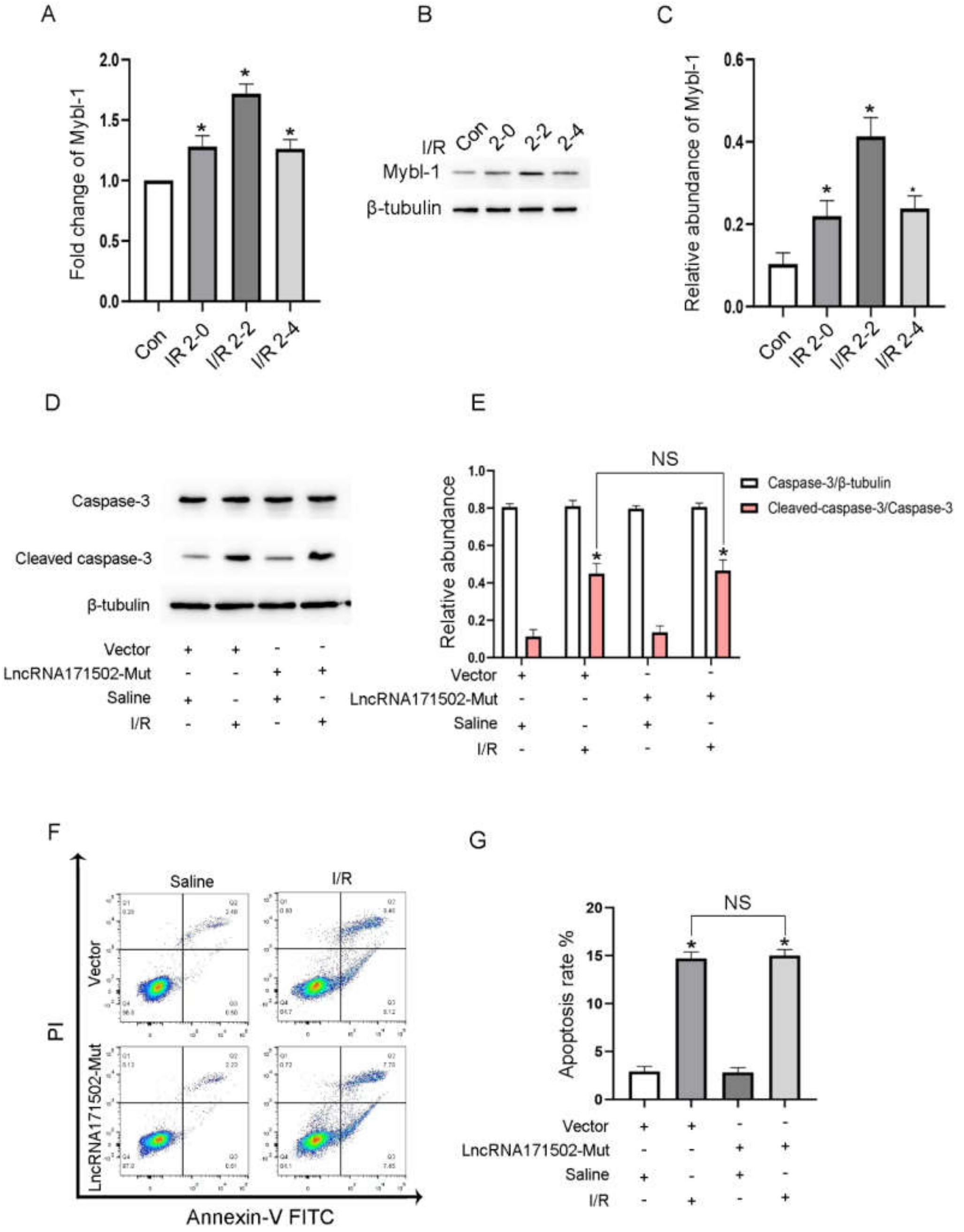

Supplementary Figure S1. The effect of I/R on the expression of Mybl-1 and LncRNA171502 mutation has no effect on ischemia induced BT cells apoptosis. (**A**) BUMPT cells were subjected to 10μM antimycin A and 1.5μM calcium (I, 2 h)/ (R, 0, 2, and 4 h). RT-qPCR was applied for the observation of Mybl-1 expression. (**B**) Immunoblot for Mybl-1 and β-tubulin. (**C**) The densitometry analyzes of Mybl-1 and β-

tubulin were presented. (**D**) BUMPT cells were transfected with LncRNA171502-MUT or vector prior to I (2 h)/R (2 h) injury or not. The expressions of apoptosis-related proteins were measured by Immunoblot. (**E**) The densitometry analyzes of cleaved-Caspase3, Caspase3, and β-tubulin were presented. (**F**) apoptosis in BUMPT cells was inspected by FCM, (**G**)and representative of apoptosis rate (%) was calculated. Data are exhibited as mean ± SD (n = 6). * $p < 0.05$, I/R group versus control or vector group; NS: no significance.
